# The Effect of Prehospital Protocol Modification during COVID-19 on First-Pass Intubation Success Rates

**DOI:** 10.1017/S1049023X25000238

**Published:** 2025-04

**Authors:** Abagayle E. Bierowski, Paul C. Comber, Alexander Kuc, Aman Shah, Gerard Carroll

**Affiliations:** 1. Department of Emergency Medicine, Thomas Jefferson University, Philadelphia, Pennsylvania USA; 2. Department of Emergency Medicine, Cooper University Hospital, Camden, New Jersey USA; 3. Cooper Medical School at Rowan University, Camden, New Jersey USA

**Keywords:** COVID-19, first-pass success, intubation, prehospital airway management, supraglottic airway

## Abstract

**Introduction:**

Many Emergency Medical Services (EMS) agencies modified their protocols during the height of the COVID-19 pandemic, particularly those involving procedures that lead to an increased risk of airborne exposure, such as intubation. In 2020, local Advanced Life Support (ALS) providers’ first-line airway management device was the supraglottic airway (SGA), and tracheal intubations (TIs) were rarely performed.

**Objective:**

This study’s aim was to investigate the potential clinical effect of this pandemic-related protocol change on first-pass TI success rates and on overall initial advanced airway placement success.

**Methods:**

This study was a retrospective prehospital chart review for all ALS encounters from a single urban EMS agency that resulted in the out-of-hospital placement of at least one advanced airway per encounter from January 1, 2019 through June 30, 2021 (*n* = 452). Descriptive statistics and chi square tests were used to evaluate data. Statistical significance was defined at *P* < .05.

**Results:**

Significantly fewer TIs were attempted in 2020 (n = 16) compared to 2019 (n = 80; *P* < .001), and first-pass TI success rates significantly decreased in 2021 (n = 22; 61.1%) compared to 2019 (n = 63; 78.8%; *P* = .047). Also, SGA placement constituted 91.2% of all initial airway management attempts in 2020 (n = 165), more than both 2019 (n = 114; 58.8%; *P* < .001) and 2021 (n = 87; 70.7%; *P* < .001). Overall first-attempt advanced airway placement success, encompassing both supraglottic and TI, increased from 2019 (n = 169; 87.1%) to 2020 (n = 170; 93.9%; *P* = .025). Conversely, overall first attempt advanced airway placement success decreased from 2020 to 2021 (n = 104; 84.6%; *P* = .0072).

**Conclusions:**

Lack of exposure to TI during the COVID-19 pandemic likely contributed to this local agency’s decreased first-pass TI success in 2021. Moving forward, agencies should utilize simulation labs and other continuing education efforts to help maintain prehospital providers’ proficiency in performing this critical procedure, particularly when protocol changes temporarily hinder or prohibit field-based psychomotor skill development.

## Introduction

During the coronavirus disease 2019 (COVID-19) pandemic, many Emergency Medical Services (EMS) agencies published new protocols or advised adaptations to their current protocols in an effort to decrease the risk of virus exposure to prehospital providers. These protocol modifications varied and included, but were not limited to, eliminating the use of nebulized medications, promoting the use of video laryngoscopy (VL) for endotracheal intubation when necessary, and minimizing the use of non-invasive positive pressure ventilation (NIPPV) when possible.^1^ The primary goal of these changes was to reduce aerosol-generating procedures, thereby mitigating the risk of viral transmission to EMS personnel. Such adaptations were crucial in maintaining the safety and health of first responders, who were at the forefront of the pandemic response and at heightened risk of exposure.

The local agency in this study implemented several similar policies, which included the preference for using supraglottic airway devices (SGAs) rather than endotracheal tubes (ETTs) as the first-line management device for respiratory failure in the prehospital setting. Due to the decrease in orotracheal intubation during the height of the COVID-19 pandemic in 2020, it was hypothesized that providers would experience lower success rates the following year as the frequency of their tracheal intubation (TI) began to increase again. The reduction in practice opportunities for orotracheal intubation during the pandemic raised concerns about potential skill degradation among providers. This study aimed to investigate the potential clinical effect of the pandemic-related protocol change on first and subsequent pass TI success rates, and on overall initial advanced airway placement success in the prehospital setting. Examining these outcomes can provide insight into the long-term impacts of the pandemic on EMS airway management practices and provider proficiency.

## Methods

This study met exemption criteria set forth by the Institutional Review Board at Cooper University (Camden, New Jersey USA). A retrospective prehospital chart review was conducted for all Advanced Life Support (ALS) encounters from a single urban EMS agency that resulted in the out-of-hospital placement of at least one advanced airway per encounter from January 1, 2019 through June 30, 2021 (*n* = 452). Most patient encounters resulting in advanced airway placement were due to cardiac arrest, though other emergencies such as respiratory failure and seizure were also included. Encounters of patients younger than 18 years old were excluded, as were encounters in which a patient’s tracheostomy tube became dislodged. All statistical analyses were performed by study investigators using statistical analysis software. Data were analyzed using odds ratios (OR) and chi square tests, then entered into a secure Microsoft Excel spreadsheet, Version 16.34 (Microsoft Corporation; Redmond, Washington USA). Statistical significance was defined at *P* < .05.

## Results

**Figure 1.**
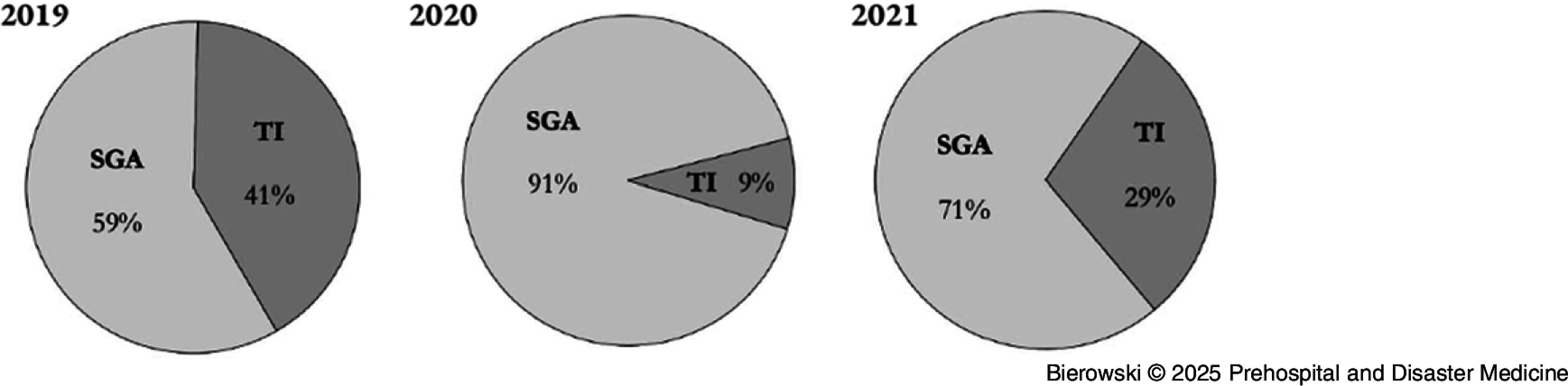
Initial Airway Management Modality. Abbreviations: SGA, supraglottic airway; TI, tracheal intubation.

Consistent with protocol modification, prehospital providers utilized SGAs as their preferred initial airway management device in 2020 (Figure 1). More supraglottic intubations (n = 165) and fewer TIs (n = 16) were attempted in 2020 compared to both 2019 (n = 114; *P* < .001) and 2021 (n = 87; *P* < .001). In 2019, two unsuccessful attempts (0.0%) at subsequent supraglottic intubation were made after initial airway placement had failed, compared to 14 of 21 successful subsequent attempts at TI (66.7%). In 2020, protocol modification prevented any subsequent attempts at placement of an SGA after an initial attempt had failed; however, ten subsequent attempts at TI had been made, with eight being successful (80.0%). In 2021, one of two subsequent attempts (50.0%) at supraglottic intubation were successful after initial airway placement had failed, while 10 of 12 subsequent attempts (83.3%) at TI were successful. Providers were 1.8-times more likely to be successful on a subsequent, rather than initial, attempt at TI in 2020 (80.0% versus 68.8%; OR = 1.82; 95% CI, 0.28-11.87) and three-times more likely to be successful on a subsequent attempt in 2021 (83.3% versus 61.1%; OR = 3.18; 95% CI, 0.61-16.73).

**Figure 2.**
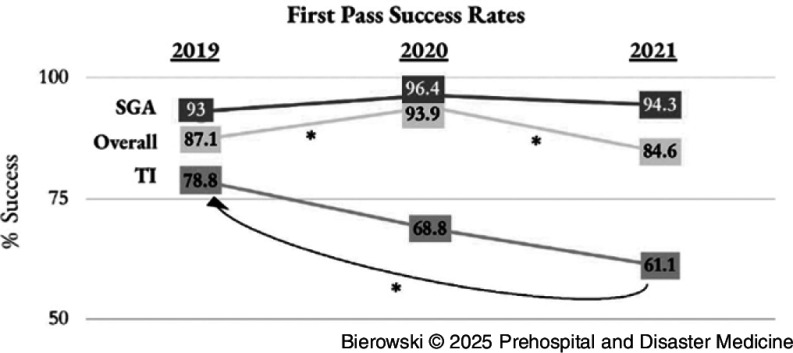
First-Pass Success Rates. Note: * Indicates *P* < .05. Abbreviations: SGA, supraglottic airway; TI, tracheal intubation.

The overall success rate of initial prehospital placement of all advanced airways (encompassing both SGA placement and TI) significantly increased in 2020 (93.9%) compared to 2019 (87.1%; *P* = .25). Conversely, overall first-attempt advanced airway placement success decreased from 2020 to 2021 (n = 104; 84.6%; *P* = .007). Though not statistically significant, first-pass success rates for supraglottic attempts marginally increased in 2020 (96.4%) compared to 2019 (93.0%; *P* = .20) and 2021 (94.3%; *P* = .44). First-pass TI success rates were lower in 2020 (68.8%) and in 2021 (61.1%) than in 2019 (78.8%; Figure 2); the decrease in first-pass TI success rates from 2019 (n = 63) to 2021 (n = 22) was statistically significant (*P* = .05), though the decreases from 2019 to 2020 (n = 11; *P* = .38) and from 2020 to 2021 (*P* = .60) were not statistically significant.

## Discussion

Guidelines for the initial emergent airway management of patients in respiratory failure were modified to some extent for nearly all emergency providers, including EMS personnel, during the COVID-19 pandemic.^1^ These guideline modifications differed from one hospital system to another and frequently changed throughout the course of the pandemic as public health officials learned more about the pathophysiology of viral infection and principles of its transmissibility. In general, guidelines recommended the utilization of rapid sequence intubation (RSI) technique utilizing VL to intubate patients, when necessary, with minimal use of bag valve mask (BVM) technique to decrease the risk of aerosolizing virus particles.^2–4^ Similarly, placement of a surgical mask over any nasal cannula and/or face mask utilized for apneic oxygenation was recommended.^3,5,6^

Just as the management of in-hospital airway decompensation changed during the height of the pandemic, EMS agencies also modified their protocols to decrease the risk of airborne exposure to the virus. Certain guidelines recommended that first responders with access to VL should engage in one attempt at endotracheal RSI, followed by SGA insertion with a second-generation device if initial endotracheal attempt was unsuccessful. To decrease the risk of repeated and unnecessary exposures to respiratory droplets, guidelines also recommended against both the use of direct laryngoscopy (DL) and repeated attempts at TI. If providers did not have access to VL and/or RSI, second generation SGAs were favored as a preferred initial airway management method; first generation SGA devices have been shown to have an inferior seal when compared to their ETT counterparts.^4,7–9^ Unfortunately, Basic Life Support (BLS) crews practicing in areas that do not permit SGA placement within their scope of practice had to utilize BVM ventilation with a tight seal and a high-efficiency particulate air (HEPA) filter while wearing appropriate personal protective equipment (PPE).^10^

Interestingly, the American College of Emergency Physicians’ (ACEP; Irving, Texas USA) Air Method Guidelines for prehospital providers taking care of patients with suspected or confirmed COVID-19 illness do not suggest a preferred initial airway management method. In fact, the guidelines encouraged providers to consider the decreased airborne transmission risk of TI compared to NIPPV, particularly in COVID+ patients with a high risk of respiratory decline who would be likely to require TI regardless of any NIPPV trial. They did not, however, mention or address the use of RSI or VL in comparison to DL.^11^

While local ALS providers do have access to VL, it had only recently been implemented at the start of the pandemic and therefore the agency recommended SGA placement as its preferred initial airway management device. Several other institutions implemented similar guidelines paralleling this recommendation, including the Maryland Institute for Emergency Medical Systems (MIEMS; Baltimore, Maryland USA), which advised the use of SGAs rather than traditional TI methods to all its EMS providers and clinicians.^12^

Though past studies have demonstrated that successful prehospital TI is associated with improved outcomes over SGA insertion, the Agency for Healthcare Research and Quality’s (AHRQ; Rockville, Maryland USA) recent systematic review of prehospital airway management data found no difference in primary patient outcome when comparing different prehospital airway management techniques across all emergency types and age groups.^13,14^ In adult and pediatric patients with cardiac arrest and adult patients with mixed emergency types, however, outcomes did favor SGA placement when comparing first-pass success rates of SGAs and ETTs.^14^

It is clear from this study that providers, even after the height of the COVID-19 pandemic, were more successful on their first attempt when placing an SGA compared to an ETT. The protocol change, prioritizing SGA placement over RSI/TI, likely contributed to the observed decrease in intubation success rates, as the lack of practice with the procedure in 2020 could have impacted provider proficiency over time. This local agency did not implement specific simulation or training sessions during the pandemic to offset this decline, which may have further affected success rates. These findings underscore an important opportunity to enhance prehospital providers’ psychomotor skill development, especially when protocol changes limit hands-on experience. Although the direct translation of simulated to real-time procedural competence remains uncertain, studies support the value of high-quality simulation in teaching and assessing skills that are infrequently performed in the field.^15–18^

Research underscores the importance of skill retention and the impact of training frequency on intubation success rates for prehospital providers. Studies, such as Carney, et al’s systematic review, indicate that SGAs generally yield higher first-pass success rates compared to TI in prehospital settings, suggesting that SGAs may serve as a more reliable option when provider exposure to ETI is limited.^19^ Similarly, Abelsson, et al highlight the critical role simulation plays in maintaining competence for complex, high-stakes procedures like intubation, which are infrequently performed in real-world settings.^20^ Without simulation or consistent hands-on training, prehospital providers may experience skill decay, impacting success rates during emergent airway management. Additionally, foundational work by Pepe, et al emphasizes that, while TI offers definitive airway control, it requires substantial initial training and periodic refreshers to maintain proficiency – resources that were challenging to provide during the COVID-19 pandemic.^21^ These insights collectively support the need for regular simulation-based training or alternative approaches, such as prioritizing SGA placement, to enhance provider readiness and mitigate the impact of procedural infrequency on patient outcomes.

From a management standpoint, awareness of staff procedure success rates is also crucial for agency leaders to identify areas of improvement and plan continuing education opportunities that address areas of weakness across agency providers. Transparency with providers regarding the results of these studies is also important, not only to their understanding of their skill development and growth as providers and teachers in the field, but also to their clinical practice. Based on feedback, one may elect to seek further agency- or local-based opportunities for improvement, or reconsider whether the benefit of proceeding with an infrequently practiced procedure outweighs the risks if unsuccessful. Further studies are required to determine the most appropriate clinical scenarios for providers to utilize SGA placement or RSI/endotracheal intubation as their first-line advanced airway management strategy, and to identify the best approach to remediation or education regarding any identified psychomotor skill deficiencies.

## Limitations

As this was a single center study based on one urban EMS agency, generalizability to other agencies in various environments is limited. Though the study’s breadth of data could also be expanded, it was inherently limited due to the duration of the COVID-19 pandemic. The accuracy and precision of data collection and entry also offers room for improvement. This study was also limited to examining aggregate first-pass success rates based on available data from a retrospective chart review, which did not include other pertinent variables such as individual performance metrics, quality assurance data, or staff turnover information. In future studies, to ensure maximum accuracy, a second blinded researcher could verify all collected data and implement a strategy to resolve any conflict(s) that arose when verifying the data coding of each encounter.

## Conclusions

Although factors such as employee turnover or individual performance could not be controlled for, it is possible that limited exposure to TI during the first year of the COVID-19 pandemic contributed to this agency’s decreased first-pass success rates in 2021. Though this agency’s decreased success rate occurred in the setting of a pandemic, this study emphasizes the validity of frequent agency review of provider success rates for life-saving procedures to ensure any deficiencies or inconsistencies can be identified and addressed. Simulation labs and other continuing education efforts may help agencies maintain provider proficiency in performing life-saving procedures, particularly when protocol changes hinder or prohibit the field-based development of key psychomotor skills.
